# GPR55 Inactivation Diminishes Splenic Responses and Improves Neurological Outcomes in the Mouse Ischemia/Reperfusion Stroke Model

**DOI:** 10.3390/cells13030280

**Published:** 2024-02-03

**Authors:** Sachin Gajghate, Hongbo Li, Slava Rom

**Affiliations:** 1 Department of Pathology and Laboratory Medicine, Lewis Katz School of Medicine, Temple University, Philadelphia, PA 19140, USA; 2 Center for Substance Abuse Research, Lewis Katz School of Medicine, Temple University, Philadelphia, PA 19140, USA; hongbo.li@temple.edu

**Keywords:** ischemia/reperfusion, CD4^+^T-cell infiltration, Tregs, spleen, stroke

## Abstract

Although strokes are frequent and severe, treatment options are scarce. Plasminogen activators, the only FDA-approved agents for clot treatment (tissue plasminogen activators (tPAs)), are used in a limited patient group. Moreover, there are few approaches for handling the brain’s inflammatory reactions to a stroke. The orphan G protein-coupled receptor 55 (GPR55)’s connection to inflammatory processes has been recently reported; however, its role in stroke remains to be discovered. Post-stroke neuroinflammation involves the central nervous system (CNS)’s resident microglia activation and the infiltration of leukocytes from circulation into the brain. Additionally, splenic responses have been shown to be detrimental to stroke recovery. While lymphocytes enter the brain in small numbers, they regularly emerge as a very influential leukocyte subset that causes secondary inflammatory cerebral damage. However, an understanding of how this limited lymphocyte presence profoundly impacts stroke outcomes remains largely unclear. In this study, a mouse model for transient middle cerebral artery occlusion (tMCAO) was used to mimic ischemia followed by a reperfusion (IS/R) stroke. GPR55 inactivation, with a potent GPR55-specific antagonist, ML-193, starting 6 h after tMCAO or the absence of the GPR55 in mice (GPR55 knock out (GPR55ko)) resulted in a reduced infarction volume, improved neurological outcomes, and decreased splenic responses. The inhibition of GPR55 with ML-193 diminished CD4^+^T-cell spleen egress and attenuated CD4^+^T-cell brain infiltration. Additionally, ML-193 treatment resulted in an augmented number of regulatory T cells (Tregs) in the brain post-tMCAO. Our report offers documentation and the functional evaluation of GPR55 in the brain–spleen axis and lays the foundation for refining therapeutics for patients after ischemic attacks.

## 1. Introduction

Stroke is not just a prominent death cause but also constitutes almost 55% of all neurological conditions, standing as the primary cause of lasting mental and physical impairments [1,2]. Although inflammation significantly contributes to ischemic stroke and reperfusion, the specific causal mechanisms are largely elusive [2,3,4,5]. The probable factor is that brain ischemia might interrupt the delicate balance between anti-inflammatory and pro-inflammatory responses, further exacerbating the activation and migration of inflammatory cells into the brain. Consequently, curbing these inflammatory reactions could reduce stroke-induced infarct size and avert neurological impairments [1,4].

Despite the high incidence and severity of stroke, options for treatment continue to be limited. Tissue plasminogen activators (tPAs), the sole FDA-approved thrombolytic agent for clot treatment, are only employed in a small subset of patients [5]. Yet, outside clot management, few methods exist for addressing the brain’s inflammatory responses to stroke. Consequently, developing alternative or complementary treatment approaches is paramount. 

Soon after an ischemic stroke followed by reperfusion (IS/R) injury, the cerebral endothelium and astrocytes generate a plethora of cytokines and chemokines, triggering sequential inflammatory cell activation and recruitment, including monocytes/macrophages, neutrophils, and T cells. Consequently, restraining these inflammatory responses diminishes infarction size and mitigates neural impairments [4]. The participation of the peripheral immune system comes into play in the later stages of neural injury development post-stroke. Extensive animal studies have effectively documented the peripheral immune cells’ intrusion into the brain after IS/R [6,7,8,9,10,11,12,13]. Despite these observations, ongoing investigations aim to unravel the definite influence of these cells on the evolution of neural injury following a stroke, primarily due to uncertainties about their origin. However, emerging evidence suggests that the spleen could be among the sources contributing to these immune cells. Regulatory T cells (Treg) are a subclass of CD4^+^ T cells that are involved in preserving self-tolerance and immune homeostasis by constraining the pro-inflammatory actions of effector T cells (both CD8^+^ and CD4^+^), antigen-presenting cells, and natural killer (NK) cells. Tregs have been shown to support self-tolerance under physiological conditions and avert the immune system’s overactivation in systemic and CNS-related inflammatory diseases, including stroke [9,14,15]. An increase in the Treg number is considered a compensatory mechanism for alleviating immune reactions in the spleen [13].

Initially identified as a responder to both natural and synthetic cannabinoids, along with endocannabinoids, G protein-coupled receptor 55 (GPR55) earned its classification as an orphan G protein-coupled receptor and a new cannabinoid receptor [16]. Yet, subsequent data converged, highlighting lysophosphatidylinositol (LPI) as the singular consistent endogenous ligand [17,18]. GPR55’s reaction to LPI finds modulation via endocannabinoids, enhancing LPI effects at lower concentrations while inhibiting them at higher levels [17]. GPR55′s expression is documented in various organs/tissues, spanning the gastrointestinal (GI) tract, CNS, spleen, and adrenal glands [17,19,20,21]. Notably, diverse leukocytes, including neutrophils, monocytes, lymphocytes, and macrophages, also exhibit GPR55 expression [22]. In contrast to conventional cannabinoid (CB) receptors, GPR55 signaling occurs due to Gq and Gα12/13 proteins [16], triggering Ca^2+^ release and the activation of downstream MAP kinases like ERK1/2, along with small G proteins such as RhoA, and activating transcription factors NFkB and NFAT [23,24]. These signaling pathways faced inactivation from the novel GPR55 inhibitor CID16020046 [20,23,24]. Notably, GPR55 diverges from classical CB receptors by inciting excitatory rather than inhibitory effects, hinting at its potential to promote functions opposing those initiated by CB receptors. Recent revelations on the interaction between CB receptors and GPR55, along with their mutual influence, underscore GPR55’s potential significance in CB receptor signaling and downstream effects [25,26]. GPR55′s involvement in inflammation has been reported [20,21,27]. GPR55 activation in vitro (rat BMVEC) resulted in endothelial cell dysfunction [21]. The activation of GPR55 with the agonist O-1602 led to the heightened production of pro-inflammatory cytokines and increased cytotoxicity in monocytes and NK cells exposed to lipopolysaccharides (LPSs) [22]. Corroborating this, when treated with an antagonist of GPR55, CID16020046 or GPR55^−/−^ knockout (GPR55ko) mice exhibited a decrease in pro-inflammatory cytokines in a colitis mouse model [28]. While the involvement of CB receptors in stroke and inflammation is well documented [29,30,31,32,33,34,35], information concerning GPR55’s role in stroke physiology remains scarce.

Animal models serve as a crucial instrument in examining human diseases. Over the last few decades, countless models mimicking stroke in animals have been extensively employed to explore ischemic mechanisms and develop drugs. Among these models, the middle cerebral artery occlusion (MCAO) model stands out as the most akin to ischemic stroke in humans and has been utilized in nearly 50% of studies of neuroprotection [36,37]. Hence, the MCAO method using intraluminal suturing in rodents gains wide recognition and standardized practice due to dependable infarct volume and its relatively simple procedure. The model of permanent cerebral ischemia mimics individuals lacking vascular recanalization, while the cerebral IS/R model mimics patients experiencing vascular obstruction followed by the prompt restoration of blood flow (recanalization). The underlying pathological mechanisms in these two models distinctly differ. Given the extensive use of the MCAO model in ischemic stroke studies, both permanent MCAO (pMCAO) and transient MCAO (tMCAO) are also employed for investigation purposes [36,37]. Since most people who suffer from cerebral clot formation undergo recanalization procedures or thrombolytic treatments (in other words, they undergo ischemia followed by reperfusion (IS/R)) [38], we decided to use the tMCAO model, an in vivo IS/R model of stroke, in this paper.

In the current study, we show that GPR55 inactivation in mice treated with the most potent specific antagonist ML-193 [20,27,28] starting 6 h after tMCAO resulted in better neurological outcomes. ML-193 (also known as CID1261822) has a specificity towards GPR55 that is greater than 145-fold, exceeding 27-fold and 145-fold when compared to GPR35, CB1, or CB2 [19,20]. These improvements were associated with decreased neuroinflammation and leukocyte infiltration. Spleen responses have been shown to be detrimental to stroke recovery [13,39,40]. Splenectomy has been demonstrated to offer neuroprotection in several stroke models and traumatic brain injury (TBI) due to a reduction in leukocyte infiltration into the brain [13,40,41]. Our data indicate that GPR55 inactivation reduced splenic responses and increased Treg’s migration into the brain. Our results point to a novel (neuro)immunomodulatory potential of GPR55.

Taking these results together, we propose that the inhibition of GPR55 confers significant neuroprotective effects following ischemia, and treatment with GPR55 antagonist(s) could represent a significant novel strategy for stroke treatments, whether administered independently or in combination with other therapeutic approaches. 

## 2. Materials and Methods

### 2.1. Animals 

Approval for all animal experiments was granted by the Temple University Institutional Animal Care and Use Committee (protocol number 4941). The experiments were carried out in adherence to Temple University guidelines, aligning with the National Institutes of Health (NIH) guide for the care and use of laboratory animals. The study’s design, experimental procedures, housing and husbandry, and statistical methods were in accordance with the ARRIVE (Animal Research: Reporting In Vivo Experiments) guidelines, available at “www.nc3rs.org.uk/arrive-guidelines (accessed on 25 January 2023)”. Male C57BL/6 mice, aged 12 weeks, were procured from the Jackson Laboratory (Bar Harbor, ME, USA). GPR55 knockout (GPR55ko) [28] mice were bred and genotyped at Temple University Animal Facilities. The mice employed in the experiments underwent at least 20 generations of backcrossing onto the C57BL/6 strain. Whenever feasible, GPR55ko and wild-type (WT) littermates were utilized in the same experiments. GPR55ko mice were matched with WT mice in terms of age. In this study, a total of 60 animals were used. 

### 2.2. Transient Middle Cerebral Artery Occlusion (tMCAO) and GPR55 Antagonist Treatment

The mice underwent 60 min of focal cerebral ischemia induced with a transient intraluminal occlusion using a 6–0 nylon monofilament with a rounded tip (Doccol Corp., Sharon, MA, USA, cat# 602312PK10) inserted into the middle cerebral artery (MCAO), following established protocols [1,42]. To accomplish tMCAO, the suture was removed while the mice were re-anesthetized. Monitoring the mouse’s body temperature is crucial throughout surgery and until it fully emerges from anesthesia. Body temperature directly influences the extent of infarction, with hypothermia reducing and hyperthermia increasing the size of the infarct [43]. Throughout surgery, body temperature was continuously monitored using a rectal probe and maintained at 37.0  ±  0.5 °C via a heating pad (Sunbeam, Neosho, MO, USA). All animals underwent a recovery period of at least 4 h on the heating pad after the procedure. Sham-operated mice underwent an identical surgical procedure, but the filament was not sufficiently advanced to occlude the middle cerebral artery. Mice were subjected to tMCAO (1 h occlusion followed by reperfusion), and 6 h after stroke onset, they were injected intraperitoneally (IP) with the highly selective GPR55 antagonist, ML-193. ML-193 was procured from Tocris (Bio-Techne Co., Minneapolis, MN, USA, cat# 4860) and prepared in DMSO at 1 μg/μL (stock solution), which was further diluted 1/1000 for injection at a dose of 1 μg/mg body weight [20,44]. Vehicle-only (0.001% DMSO) injected mice were used as controls. Treatment was repeated 24 and 48 h post-stroke. At 72 h post-MCAO induction, mice were anesthetized with 5% isoflurane to minimize pain and distress. Subsequently, they were euthanized through cervical dislocation and decapitated, and their brains were collected. Rigor in tMCAO experiments was guaranteed by monitoring each mouse for regional cerebral blood flow (rCBF) before ischemia, during MCAO, and after reperfusion using a Laser Speckle PeriCam PSI System (Perimed AB, Järfälla, Sweden). If the rCBF did not decrease to at least 25% of the initial level, the animal was excluded from the study and euthanized [34]. Mice were excluded from subsequent studies if (a) there was excessive bleeding during surgery, (b) the mice did not recover from anesthesia within 15 min, or (c) the mice did not survive the 72-h period after the onset of stroke.

### 2.3. Neurological Assessment

Each mouse underwent a series of behavioral assessments 3, 24, 48, and 72 h after tMCAO. Benderson’s (0–5) score was used in our laboratory [45,46,47], and it was performed 3 h and 24 h after the induction of ischemia, and scored as follows: 0, no neurological deficit; 1, failure to extend left forepaw fully; 2, constant circling to the left; 3, falling to the left; 4, no spontaneous walking. Mice that scored 0 or 4 were excluded from the studies. A global neurological amelioration was witnessed in augmented locomotor activity (assessed with overall ambulation counts) [48,49,50,51] at 24, 48, and 72 h. The corner test, designed to detect sensorimotor and postural symmetries, was performed at 72 h post-stroke. “Stroked” mice typically exhibit a turning preference toward the affected side (right), whereas unaffected mice display an almost equal distribution of left and right turns, approximately 50–50%. During the corner test, all mice were permitted to enter a corner angled at 30°, necessitating a turn either to the right or the left for exiting. This procedure was repeated and recorded ten times, with a minimum interval of 30 s between trials. The percentage of right turns out of the total turns was then calculated [51,52,53].

### 2.4. Preparation and Collection of Brain for 2,3,5-Triphenyltetrazolium Chloride (TTC) Staining

TTC staining serves as a marker for metabolic activity, reliably indicating ischemic areas in investigational models of stroke. This colorless water-soluble dye is processed by the succinate dehydrogenase enzyme in mitochondria in viable cells, transforming it into water-insoluble formazan, a light-sensitive compound that imparts a deep red hue in healthy tissue. In contrast, damaged or diseased tissue retains its white color, signifying the lack of alive cells and distinctly marking the infarcted area [45,54]. On day 3 post-stroke, mice were anesthetized deeply using an isoflurane 5% (Sigma-Aldrich Cat# 792632, St. Louis, MO, USA). Animal perfusion was accomplished transcardially using ice cold 0.9% NaCl (Fisher Scientific, Cat# Z1376) for 3 min. Following that, brains were dissected and sectioned into 2 mm coronal slices from the olfactory bulb to the cerebellum using a matrix device (Zivic Instruments #5325, USA). For brains treated with TTC (n = 5), sections were immersed in a 2% TTC solution (Sigma-Aldrich #T8877) in saline and incubated at 37 °C for 10 min [54]. The TTC-treated brain sections were photographed using a digital camera (Sony HDR-PJ790, Sony Corp., Tokyo, Japan) for subsequent analysis of infarction size, with the infarct area recognized as the none-stained region using TTC in stroke mice and the corresponding contralateral section [54].

### 2.5. Spleen Measurements and Splenocyte Isolation

Three days after the stroke, mice were euthanized as described above, and spleens were removed and cleaned from fat tissue. Spleen size was measured, and splenocytes were isolated via the dilaceration of the spleen with a syringe piston above a 70 m cell strainer, followed by subsequent treatment with a red blood cell lysis buffer (eBiosciences, San Diego, CA, USA) and washed with an ice cold FACS staining buffer containing 1xPBS/2%FBS (eBiosciences) [55].

### 2.6. Flow Cytometry (FACS) for Brain Infiltrating Leukocytes and Splenocytes

Brain-infiltrating leukocytes (BILs) were separated from other brain cells via Percoll/Ficoll centrifugation, both from non-infarcted and infarcted hemispheres [46,56,57]. To identify lymphocytes coming from the spleen, mice were injected with 5(6)-carboxyfluorescein diacetate succinimidyl ester (CFSE) into their spleens [58]. In short, 25 mg of CFSE (eBiosciences, cat#65-0850-84) dissolved in 4 mL of dimethyl sulfoxide, including 40 μL of heparin (1000 U/mL), was injected into the spleen under anesthesia, with a volume of 100 μL. BILs and splenocytes were surface-stained with antibodies against mouse CD45 (clone 30-F11), CD11b (clone M1/70), CD3 (clone 500A2), CD4 (clone GK1.5), and CD25 (clone PC61), which were all purchased from BioLegend (San Diego, CA, USA), at 4 °C for 30 min. The IC fixation buffer (eBiosciences, San Diego, CA, USA) was utilized to fix the cells. Cytometric acquisition was accomplished using an Aurora flow cytometer (Cytek Biosciences, Fremont, CA, USA) and analyzed using FlowJo software v.10 (Tree Star, Inc., Ashland, OR, USA).

### 2.7. Statistical Analysis

Data are expressed as the mean ± SD. Behavioral tests were performed at least 3–4 times on each mouse, and flow cytometric assessments were performed in duplicate for each mouse. On the graph, each symbol represents the average for each mouse. The data underwent normality testing using the Shapiro–Wilk test. If the data exhibited a normal distribution, one-way ANOVA was conducted for multiple group comparisons, followed by the Tukey post hoc test, with significance set at *p* < 0.05 (for FACS and animal experiments). Statistical analyses were carried out using Prism v10 software (GraphPad Software Inc., San Diego, CA, USA).

## 3. Results

### 3.1. GPR55 Inactivation Alleviates the Neurological Outcomes of tMCAO and Reduces Brain Infarction

**Figure 1:**
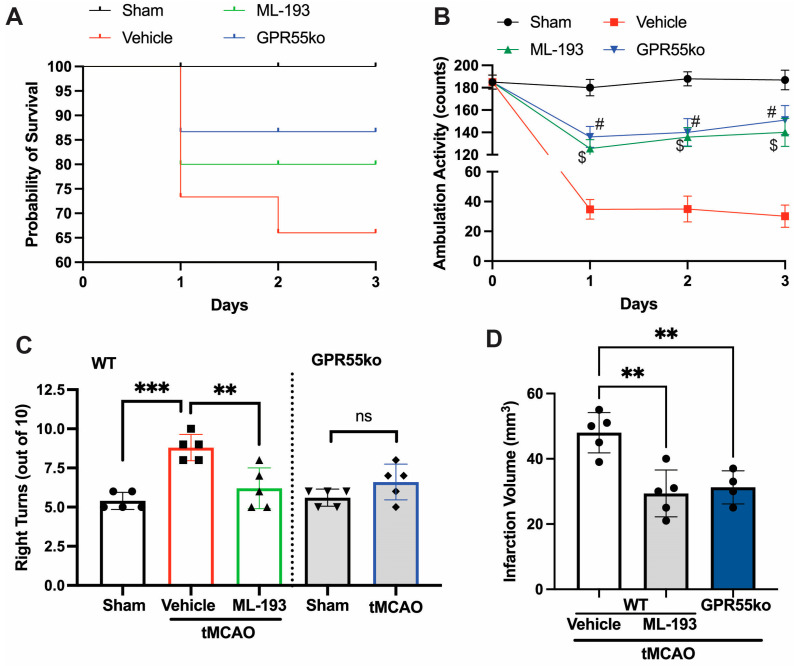
GPR55 inactivation improves neurological scores and reduces the infarction area in tMCAO. Mice were subjected to tMCAO and treated with GPR55 antagonist (ML-193) at a dose of 1 μg/kg body weight, starting 6 h after tMCAO and then every 24 h. Vehicle only (0.001% DMSO in sterile PBS) was injected as a negative control. Sham-operated animals were also injected with vehicles only as negative controls. GPR55ko mice were subjected to tMCAO and assessed as wild-type (WT) mice. (**A**) Survival curve. Total ambulation activity (**B**) and corner tests (**C**) were acquired at 3 days following ischemia reperfusion. (**D**) The infarction area was assessed by measuring the affected area after TTC staining of the brains. Results are represented as mean ± SD from 5 animals per each condition. One-way ANOVA was performed with the Tukey post hoc test to evaluate significance. ** *p* < 0.01, *** *p* < 0.005. $ or # represents *p* < 0.05 vs. vehicle-injected tMCAO mice group. ns—non-significant.

Ischemic stroke arises from a temporary or enduring decline in regional blood flow. In humans, the predominant occurrence of ischemic stroke takes place in the region perfused by the middle carotid artery (MCA) [1,4]. The stroke models of utmost relevance involve permanent or transient MCA occlusion (pMCAO or tMCAO) [4,45,59]. Emerging data suggest that the tMCAO model may better imitate the pathophysiology of stroke in humans compared to pMCAO [1,4]. We hypothesized that the inhibition of GPR55 activation would reduce neuroinflammation caused by IS/R and restore neurological functions. With this goal in mind, mice were subjected to tMCAO (1 h occlusion followed by reperfusion), and 6 h after stroke onset, they were injected with IP and 1 μg/mL ML-193, the most potent GPR55 antagonist, or with the vehicle only (0.001% DMSO) as a control, respectively [20,44]. Inhibition with selective antagonist ML-193, as well as GPR55 absence (in GPR55ko), improved Bederson’s neurological scores by 60% (at day 1 after stroke, not shown) and improved survival rates post-tMCAO (Figure 1A). tMCAO caused a ~5.7-fold reduction in locomotor activity on days 1, 2, and 3 post-IS/R (Figure 1B). Almost 87% of neurological enhancement was observed in greater locomotor activity (tested with overall ambulation counts) [48,49,50,51] in GPR55ko mice and animals treated with ML-193 (Figure 1B). To observe whether GPR55 inactivation would show long-lasting neurological improvements, we performed the corner test 3 days after the IS/R event. “Stroked” mice generally tend to turn toward the stroke-affected side (right), while non-disturbed mice have an almost equal left-to-right turn allocation [46,51,52,60]. Indeed, mice treated with the specific GPR55 antagonist, ML-193, showed 92% ± 3% recovery in the corner test (*p* < 0.01) (Figure 1C). The absence of GPR55 resulted in a near-full recovery in the corner test (Figure 1C). 

Treatment with a specific GPR55 antagonist, ML-193, resulted in a 34% ± 5% improvement in the infarction area (Figure 1D), measured via the TTC stain [45,46,47,54]. Similar results were observed in GPR55ko animals (Figure 1D). 

These findings indicate that the deactivation of GPR55 may be administered as early as 6 h after the onset of stroke to mitigate neurological impairments.

### 3.2. GPR55 Inactivation Attenuates Spleen Size Reduction and Reduces CD4^+^T-Cell Egress from the Spleen following tMCAO in Mice

**Figure 2:**
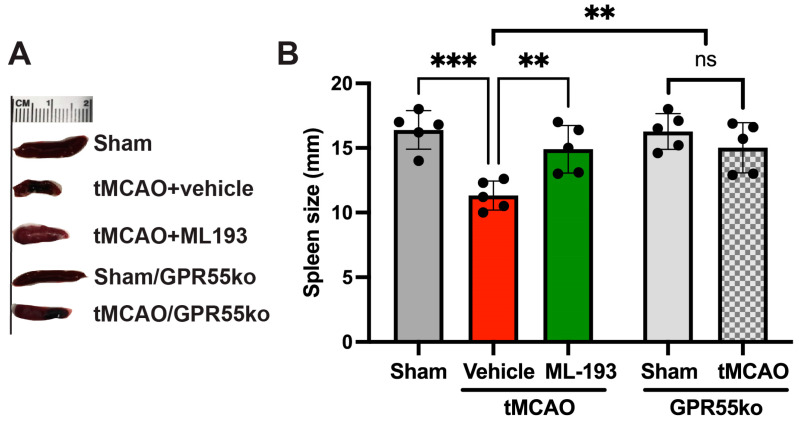
Inactivation of the GPR55 receptor decreases spleen contraction after stroke. Mice were subjected to tMCAO and treated with GPR55 antagonist (ML-193) at a dose of 1 μg/kg body weight starting 6 h after tMCAO and then every 24 h. Vehicle only (0.001% DMSO in sterile PBS) was injected as a negative control. Sham-operated animals also were injected with vehicles only as negative controls. GPR55ko mice were subjected to tMCAO and assessed as WT mice. Spleens were isolated on day 3 post-IS/R, and their size was analyzed (n = 5). (**A**) Representative pictures. (**B**) Quantitative representation of spleen size. Data are displayed as mean ± SD. One-way ANOVA was performed with the Tukey post hoc test to evaluate significance. ** *p* < 0.05, *** *p* < 0.01. ns—non-significant.

The splenic reaction to stroke involves a pro-inflammatory reaction to IS/R injury, leading to increased neurodegeneration. A spleen size reduction after stroke has been shown both in humans and animals [12,61,62,63,64]. We decided to check whether GPR55 inactivation will prevent spleen size reduction. Spleen size was measured at 3 days post-stroke in mice, and indeed, tMCAO led to a 32.6% reduction in spleen size, whereas treatment with ML-193, a specific GPR55 antagonist, diminished these effects by almost 93% (Figure 2). Comparable results were witnessed in GPR55ko mice at 3 days after tMCAO (Figure 2).

These results point out the capability of GPR55 inactivation in reducing splenic responses after stroke.

### 3.3. GPR55 Inhibition Weakens Inflammatory Reactions upon IS/R Conditions In Vivo

**Figure 3:**
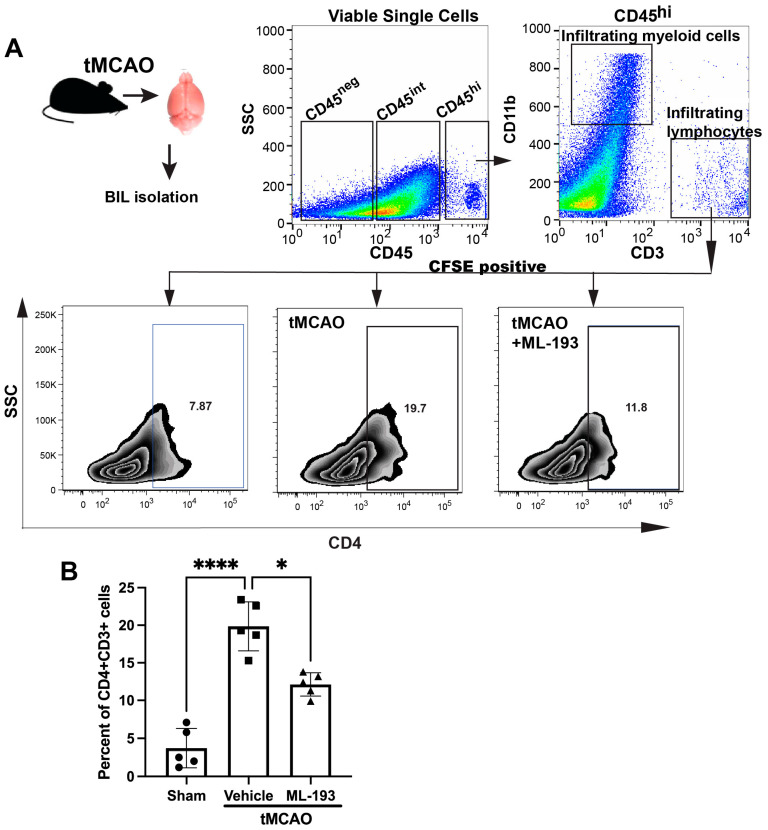
GPR55 inactivation diminishes CNS infiltration by CD4^+^T cells. Mice were subjected to tMCAO and were treated with GPR55 antagonist (ML-193) at a dose of 1 μg/kg body weight starting 6 h after tMCAO and then every 24 h. The vehicle only (0.001% DMSO in sterile PBS) was injected as a negative control. Sham-operated animals were also injected with vehicles only as negative controls. Mice were injected with CFSE into the spleens to stain splenocytes. BILs were isolated on day 3 post-IS/R, stained with fluorescently labeled antibodies, and analyzed via FACS. Flow cytometry examination of BILs isolated from infarcted hemisphere (n = 5) was performed. In total, 10,000 events were recorded per tube. (**A**) Gating strategy and representative contour plots analysis for CD45^hi^CD3^+^ lymphocytes. (**B**) Quantitative representation of T-cell populations. Data are represented as mean ± SD. One-way ANOVA was performed with the Tukey post hoc test to evaluate significance. * *p* < 0.05 and **** *p* < 0.001.

The IS/R process triggers a sequential influx and activation of inflammatory cells, including neutrophils, T cells, and monocytes/macrophages, over time. Inhibiting these inflammatory reactions diminishes the size of the infarct and ameliorates neurological impairments [1,4]. The compromised and activated blood–brain barrier (BBB) facilitates the entry of peripheral inflammatory cells into the brain, secreting harmful mediators that cause sustained barrier injury. Our working hypothesis posited that GPR55 inactivation would reduce inflammatory responses after IS/R. CD4^+^T cells [CD45^hi^CD11b^−^CD3^+^CD4^+^] recently have been shown to migrate into the ischemic brain 1–3 days post-stroke in the tMCAO model in mice [46]. Mice spleens were injected with CFSE to track T cells coming from the spleen. Next, we isolated brain-infiltrating leukocytes (BILs) from mice 3 days post-tMCAO and uncovered a ~4.7-fold amplified existence of splenic-origin CD4^+^T cells in the infarcted hemisphere (Figure 3). Treatment with ML-193 led to a ~42% ± 3.4% (*p* < 0.05) decrease in the number of splenic T cells 3 days after tMCAO induction (Figure 3B).

These data indicate that inactivation of GPR55 could prevent post-stroke CD4^+^T-cell brain infiltration and reduce neuroinflammation.

### 3.4. GPR55 Inhibition Diminishes T-Cell Egress from the Spleen

**Figure 4:**
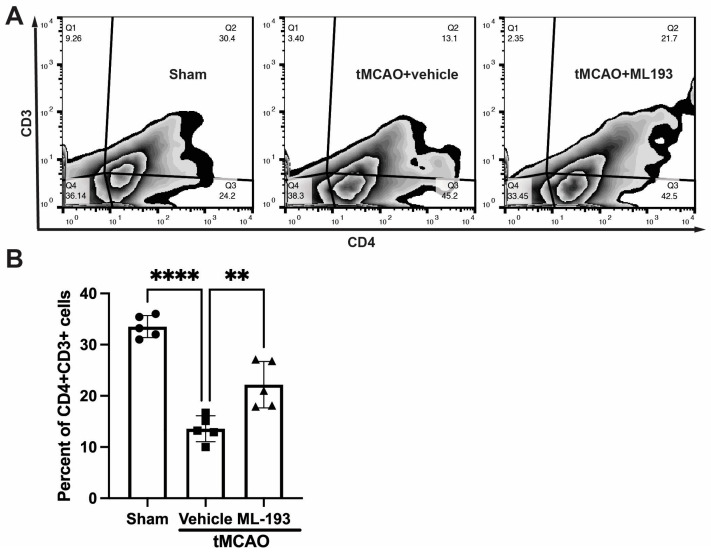
GPR55 inactivation diminishes CD4^+^T-cell egress from the spleen after stroke. Mice were subjected to tMCAO and were treated with a GPR55 antagonist (ML-193) at a dose of 1 μg/kg body weight starting 6 h after tMCAO and then every 24 h. Vehicle only (0.001% DMSO in sterile PBS) was injected as a negative control. Sham-operated animals also were injected with vehicles only as negative controls. Mice were injected with CFSE into the spleens to stain splenocytes. Splenocytes were isolated from the extracted spleens on day 3 post-IS/R, stained with fluorescently labeled antibodies, and analyzed via FACS. Flow cytometry examination of T cells isolated from spleens (n = 5) was performed. In total, 10,000 events were recorded per tube. (**A**) Representative contour plots analysis for CD45^hi^CD3^+^ lymphocytes. (**B**) Quantitative representation of T-cell populations. Data are represented as mean ± SD. One-way ANOVA was performed with the Tukey post hoc test to calculate significance. ** *p* < 0.01 and **** *p* < 0.001.

To track changes in T-cell populations in spleens, mice were injected with CFSE into their spleens following stroke, and 3 days later, splenocytes were isolated, stained with fluorescently labeled antibodies for CD4 and CD3, and analyzed via FACS. Stroke resulted in an almost 3-fold decrease in CFSE-positive CD4^+^CD3^+^T cells, whereas treatment with ML-193, a GRP55 antagonist, attenuated T-cell egress from the spleen by 62% ± 7% (Figure 4).

These results indicate the capability of GPR55 inactivation in reducing splenic responses after stroke.

### 3.5. GPR55 Inactivation Increases Treg’s Numbers in the Brain

**Figure 5:**
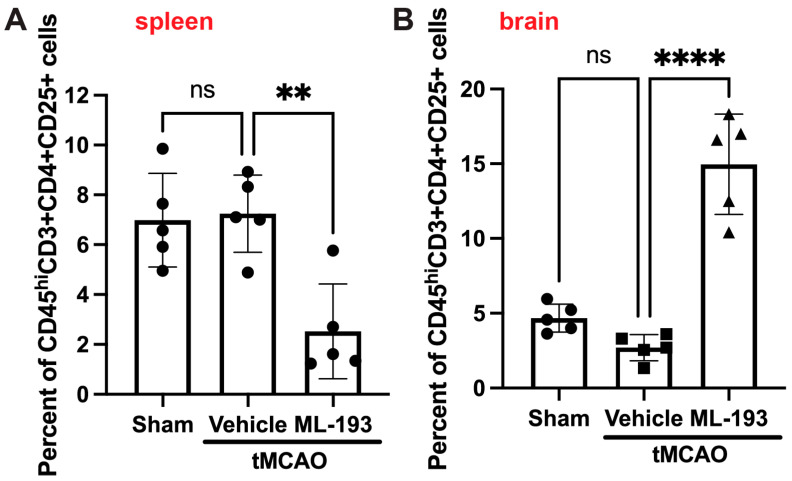
GPR55 inhibition increases Tregs’ brain infiltration after stroke. Mice were subjected to tMCAO and treated with GPR55 antagonist (ML-193) at a dose of 1 μg/kg body weight starting 6 h after tMCAO and then every 24 h. The vehicle only (0.001% DMSO in sterile PBS) was injected as a negative control. Sham-operated animals were also injected with vehicles only as negative controls. CFSE was injected into the spleens to stain splenocytes. BILs were isolated from brains, and splenocytes were isolated from the extracted spleens, stained with fluorescently labeled antibodies, and analyzed via FACS (n = 5). In total, 10,000 events were recorded. Quantitative representation of CFSE-positive Treg (CD45^hi^CD3^+^CD25^+^) populations in the spleens (**A**) and the brains (**B**). Data are represented as mean ±SD. One-way ANOVA was performed with the Tukey post hoc test to evaluate significance. ** *p* < 0.01 and **** *p* < 0.001. ns—non-significant.

Tregs play a protective role in managing post-stroke inflammation [7,9,65,66]. There have been observations of a decrease in the Tregs–whole-T-cell ratio in both humans and mice, along with reduced levels of cytokines IL-10 and TGF-β, secreted by Tregs [67]. Thus, augmenting Tregs might hold promise as a stroke treatment option. We decided to check if GPR55 inhibition could affect Treg numbers in post-stroke brains. BILs were isolated as described above and stained with fluorescently labeled antibodies against CD45, CD3, CD4, and CD25 (Treg markers accepted in the field) [6,7,8,9]. Indeed, ML-193 treatment increased ~4.7-fold the amount of Tregs in the mouse brain 3 days post-stroke, concurrently reducing their amount in the spleens by 67% (Figure 5). 

These outcomes display the capability of GPR55 inactivation in decreasing neuroinflammation by increasing brain Treg infiltration.

Collectively, our findings indicate that GPR55 inactivation improves neurological functions by reducing splenic responses, thus attenuating T-cell brain infiltration, and increasing Treg migration into the brain.

## 4. Discussion

Stroke is an overwhelming cardiovascular/neurological disease that is a prominent foundation of long lasting physical and mental disability [2,4,68,69]. About three-quarters of individuals who have survived a stroke experience difficulty in walking, and even with rehabilitation endeavors, 25% endure gait abnormalities that hinder their daily activities and mobility [70,71,72]. tPA is the sole FDA-approved thrombolytic agent for addressing thrombosed vessels, yet its application is limited to a small portion of patients due to a narrow treatment window of up to 3 h, along with notable side effects such as the potential conversion of an ischemic stroke to a hemorrhagic one and several contraindications. Furthermore, tPA fails to provide protection against reperfusion injury, which stems from the inflammation initiated in the affected blood vessels at the site of the stroke. Consequently, the development of fresh alternatives or supplementary treatment approaches for strokes is crucial.

A crucial part in the pathogenesis of IS/R stroke is played by inflammation [2,3,4,5]. IS/R-induced inflammation leads to the dysfunction of the cerebral endothelium, which originates from vascular compromise. Soon after brain ischemia, the endothelium and glia generate chemokines and cytokines. Such molecules (interleukins and TNFα) incite adhesion molecule expression on the endothelium, leading to the adhesion of leukocytes and the breakdown of the extracellular matrix and endothelial tight junction proteins (TJPs) [73,74,75]. IS/R induces the time-dependent recruitment and activation of inflammatory cells (neutrophils, T cells, and monocytes/macrophages). The impaired and inflamed BBB fosters the peripheral inflammatory cells’ migration into the brain, secreting harmful mediators and causing long-lasting BBB damage, while the inhibition of inflammatory responses reduces infarct size and improves neurological deficits [1,4]. Thus, the inhibition of inflammation and immune responses and the diminution of leukocyte infiltration would provide a comprehensive innovative therapeutic strategy for CNS protection against IS/R injury. 

GPR55, an orphan G protein-coupled receptor, is expressed not only prominently in the CNS but also in peripheral tissues and immune cells [22]. GPR55 activation and contribution to inflammation have been reported [20,21,27,76]; however, its function in stroke remains to be discovered. In this report, our results indicate that the GPR55 antagonist, ML-193, applied 6 h after stroke induction safeguarded against cerebral ischemia reperfusion injury. GPR55 inactivation noticeably attenuated the after-stroke decrease in spleen size and decreased CD4^+^T-cell spleen egress in tMCAO mice. We also found that GPR55 inactivation diminished CD4^+^T-cell infiltration in the brain, which was accompanied by diminished brain infarction and neurological outcome improvements. Following an ischemic stroke, the spleen undergoes activation, influencing both systemic inflammation and the inflammatory environment within the brain. Animal studies have detailed a biphasic cycle of brain–spleen cell interaction post-stroke. Initially, there is a pro-inflammatory phase where the spleen contracts within 1–4 days, coinciding with an elevated release of lymphocytes and monocytes into the bloodstream [11,61,63,64]. A similar biphasic spleen cycle was shown in humans post-stroke [62] and post-TBI [77]. Spleen responses have been demonstrated to hinder stroke recovery [13,39,40]. Studies indicate that a splenectomy has exhibited neuroprotective effects across various stroke and TBI models by reducing the infiltration of leukocytes into the brain [13,40,41]. These findings suggest that the inactivation of GPR55 safeguards the ischemic brain via the peripheral immune system.

The inflammatory response, governed by both the innate and adaptive immune systems, exacerbates brain damage [10]. The innate immune response primarily involves macrophages, originating from either the resident microglia or migrated peripheral monocytes. Meanwhile, the adaptive immune response is controlled by T cells and B cells. Among the leukocytes infiltrating the damaged brain from the periphery, T cells have consistently emerged as a dominant subgroup impacting secondary neurodegeneration and influencing the extent of ischemic brain damage [9]. Different T-cell subsets possess the capacity to either safeguard or exacerbate post-stroke neuroinflammation. Notably, pro-inflammatory TH1 and TH17 subsets of helper T cells, alongside IL-17-producing γδ T cells, have been observed to prompt secondary neurotoxic effects, leading to enlarged infarctions and poorer functional outcomes [78]. Among T cells, both CD4^+^ and CD8^+^T cells have been documented, and recent research suggests that T cells also impact brain damage triggered by strokes by infiltrating the ischemic brain [79]. Studies by Hurn et al. showed that deficiency in T cells notably reduced infarct size post-stroke [80]. Similar reductions in infarct size were observed in our studies and others when CD4^+^ or CD8^+^T cells were absent, as measured 1–4 days post-stroke [10,46]. The current report shows the ability of the GPR55 antagonist to reduce T-cell brain infiltration 3 days after tMCAO, which also coincided with a decrease in infarct size. T cells influence endothelial cells using these adhesion factors and traverse the compromised BBB to enter brain tissue. This migration seems to be orchestrated by interactions of p-selectin and p-selectin glycoprotein ligand 1 (PSGL1), and chemokines [9,46,81,82,83,84,85,86]. The effects of GPR55 inactivation on BBB permeability, endothelial function, and the chemokine profile post-stroke will be explored in future studies.

Conversely, regulatory T cells (Tregs) showcase anti-inflammatory and neuroprotective traits by curbing an excessive inflammatory response to the brain infarct [9]. Liesz and colleagues reported that the depletion of the CD25^+^ T-cell population with an anti-CD25 mAb significantly boosted brain infarct volume and worsened functional outcomes after transient cerebral ischemia in mice [87]. Our data demonstrate that the GPR55 antagonist ML-193 significantly increased Treg numbers in the brain 3 days post-stroke. Tregs primarily oversee the control of effector T lymphocyte function in the periphery, suppressing the activities of effector lymphocytes. This regulation sustains immune tolerance towards self-antigens and averts excessively robust reactions relative to harmless foreign antigens. Benakis and colleagues recently established a mechanistic connection between T cells and morphology, as well as the transcriptomic profile of microglia in the stroke context [88]. They demonstrated the distinct impact of various T-cell subtypes in influencing the polarization state of microglia following a stroke. Transcriptomic analyses suggest that the influence of different helper T-cell subgroups on microglial polarization predominantly occurs via their specific cytokine and chemokine secretion patterns [88]. Microglia interacting with TH1 cells displayed an elevation in type I INF-associated genes and signaling, the primary cytokine released by TH1 subtypes. Conversely, Treg cells influenced a gene profile in microglia linked to chemoattraction-related mechanisms (Ccl7, Ccl2, and Cxcl10). It remains to be elucidated whether Treg-primed microglia contribute to recruiting other immune cells, particularly those inducing neuroprotective mechanisms. Previous research underscores the direct role of IL-10, mediated by Treg cells, in modulating microglial function [88]. The activation of GPR55 has been shown to decrease IL-10 expression [76]; thus, its inactivation might reverse this effect and play a role in Treg-mediated microglia-modulating function via IL-10. Interestingly, in our hands, the stroke itself did not affect Treg number in the brain, nor in the spleen, when compared to sham-operated animals. Furthermore, investigations are required to pinpoint the complicated mechanisms in Treg migration into the brain or out of the spleen. Growing evidence supports the advantageous impact of Tregs in promoting neuroprotection and aiding in the recovery process post-stroke. This has led to investigations aimed at enhancing both the quantity and efficacy of Tregs for stroke therapy [89]. Despite their low numbers in circulation, Tregs can interact with various peripheral immune cells (e.g., effector T cells, macrophages, and neutrophils) [9,87] and resident CNS cells (e.g., microglia and astrocytes) [88], working to restore the immune environment and foster a reparative setting within the ischemic brain. An increase in Treg number is considered a compensatory mechanism for alleviating immune reactions in the spleen [13]. In this study, utilizing experimental stroke models, we have shown that augmenting Treg numbers following a stroke—via GPR55 inactivation—facilitates white matter restoration and enhances functional recovery. 

Our findings reveal a newfound potential for GPR55 in (neuro)immunomodulation. Despite the prevalence and severity of stroke, the available treatments remain restricted. However, apart from managing clots [5], limited strategies address the brain’s inflammatory reactions triggered by stroke. Hence, the development of alternative or supplementary treatment methodologies is crucial. Immune system disbalance after IS/R is induced by both peripheral immune suppression and central neuroinflammation. Immune system activation arises from the center to the periphery and then from the periphery back to the center. Investigating the mechanisms of neuroinflammation, especially the role of the spleen–brain dialogue, could provide new methods for amending cell death secondary to stroke. Considering these outcomes collectively, we propose that inhibiting GPR55 yields substantial neuroprotective effects post-ischemia. Consequently, employing GPR55 antagonists could serve as a pivotal new avenue for stroke therapies, either independently or in combination with other treatment modalities.

## Data Availability

Data are stored in the Temple University Lab Archives and are available upon request.
